# Timing of surgery in patients with synchronous colorectal cancer liver metastases undergoing neoadjuvant chemotherapy: a propensity score analysis

**DOI:** 10.1186/s12957-023-03162-y

**Published:** 2023-09-01

**Authors:** Yaoqun Wang, Ningyuan Wen, Xianze Xiong, Jiong Lu, Bei Li, Nansheng Cheng

**Affiliations:** 1 Division of Biliary Tract Surgery, Department of General Surgery, West China Hospital, Sichuan University, Chengdu, 610041 Sichuan China; 2 Research Center for Biliary Diseases, West China Hospital, Sichuan University, Chengdu, 610041 Sichuan China

**Keywords:** Colorectal cancer liver metastases, Hepatectomy, Neoadjuvant chemotherapy, Propensity score matching, Prognosis

## Abstract

**Background:**

The optimal timing of surgery after neoadjuvant chemotherapy (NAC) in patients with synchronous colorectal cancer liver metastases (SLM) remains controversial. We plan to analyze whether the choice of different surgical timings will have different effects on the perioperative and oncologic outcomes of patients.

**Method:**

We retrospectively collected all patients who met the inclusion and exclusion criteria from 2010 to 2020 in West China Hospital. Patients were grouped according to time interval (TI) after NAC to surgery. The perioperative and oncologic outcomes of the two groups were compared after propensity score matching. Univariate and multivariate analyzes were used to screen factors associated with prognosis.

**Result:**

Among 255 enrolled patients, 188 were matched with comparable baseline (94 each group). Patients in the 6≦TI≦8 group had longer operation time, less intraoperative blood loss, and less postoperative complications than those in the 4≦TI < 6 group. However, the overall survival (OS) (*p* = 0.012) and disease-free survival (DFS) (*p* = 0.013) of the patients in the 4≦TI < 6 group were better than those in the 6≦TI≦8 group. Subgroup analysis found that the above conclusions still apply in age ≥ 60, non-anemic patients, and patients who underwent R0 resection. OS was inversely correlated with TI in patients without preoperative jaundice. DFS was negatively correlated with TI in patients with preoperative jaundice. Multivariate analysis showed that the prolongation of TI after NAC to surgery was an independent prognostic risk factor for OS and DFS.

**Conclusions:**

Patients with SLM may be a better choice for surgery within 4–6 weeks after receiving NAC. Although patients with SLM undergoing surgery 4–6 weeks after NAC has a higher rate of postoperative complications, radical surgery is still recommended for a better survival benefit.

**Supplementary Information:**

The online version contains supplementary material available at 10.1186/s12957-023-03162-y.

## Introduction

Due to the anatomical characteristics of the colorectal, the liver is the most common site of blood-borne metastasis [1, 2]. About 50% of patients will develop colorectal cancer liver metastasis (CRLM) within 3 years after colorectal cancer (CRC) diagnosis, and 15 ~ 25% of patients will develop synchronous colorectal cancer liver metastases (SLM) at the time of first diagnosis [3]. The prognosis of SLM is worse than that of metachronous liver metastasis (MLM) [4, 5]. Even with therapeutic intent of resection, the prognosis of patients cannot be significantly improved due to the possibility of small metastatic foci that may not be detected by CT/MRI.

Neoadjuvant chemotherapy (NAC) is a systemic treatment for malignancies given before surgery. It is suitable for a wide range of patients and can be used as a window to identify and control metastatic lesions and guide subsequent treatment. For SLM patients, NAC can eliminate small metastatic foci before surgery, thereby minimizing the possibility of liver metastasis recurrence after curative surgery, extending recurrence-free survival (RFS) or overall survival (OS) [6]. It can also help reduce the size of existing tumor lesions, increase the chance of R0 resection, and increase the residual liver volume after surgery [7]. Currently, there is clear evidence that surgery can be scheduled 4 weeks after the last NAC [8, 9], but the optimal timing for surgery after 4 weeks is still controversial. For example, some studies have shown that appropriately extending the interval between NAC and surgery can increase the rate of tumor downstaging and the rate of pathological complete response (pCR) [10, 11]; however, prolonging the interval between NAC and surgery may increase the difficulty of surgery and reduce the quality of surgery.

In this study, we include SLM patients who received NAC treatment in our hospital and divide them into groups according to the length of the chemotherapy-surgery interval. We will use a 1:1 propensity score matching (PSM) analysis to minimize bias from nonrandom assignments [12]. We will analyze whether choosing different timing of surgery will have different effects on perioperative complications and prognosis of patients.

## Materials and methods

### Inclusion and exclusion criteria

We retrospectively collected patients with synchronous colorectal cancer and hepatic metastases who underwent simultaneous resection at West China Hospital from January 2010 to December 2020. All patients received NAC prior to surgery. This study was approved by the Ethics Committee of the West China Hospital of Sichuan University (approval No. 2022–1866).

Patients who meet the following criteria were included: (1) patients whose liver metastases found before or at the time of CRC diagnosis, (2) patients received NAC 4–8 weeks prior to operation, (3) patients underwent simultaneous resection of primary tumors and metastases, and (4) CRLM confirmed by pathological examination. Patients meeting the following criteria were excluded: (1) history of any other primary malignancy except CRC, and (2) patient who has received other adjuvant treatment besides NAC before surgery, such as radiotherapy, interventional embolization.

### Neoadjuvant chemotherapy program

Since the vast majority of patients underwent surgical resection within 8 weeks, we screened patients who underwent surgical treatment within 4–8 weeks after completing NAC for further study. For the selection of treatment cycles for patients receiving NAC treatment, it is recommended that NAC should not exceed 6 cycles in order to minimize the damage of chemotherapy drugs to the liver [13]. In our study, the choice of the actual treatment cycle of NAC was based on the clinical evidence mentioned above and was determined according to the clinical tolerance and treatment effect of the patients. Currently, the internationally recommended NAC regimens for preoperative CRLM include FOLFOX, FOLFIRI, CapeOX, or FOLFOXIRI [14]. The doctor would select the best chemotherapy regimen for patients receiving NAC treatment according to their individual conditions.

### Date collection and follow-up

Data on patient demographics, preoperative assessment, history of NAC, and operation-related variables were retrospectively collected. The pathological clinicopathological characteristics of cancer were determined by paraffin sections. All included iGBC were histopathologically confirmed by experienced pathologist. Various complications occurred during hospitalization were divided according to Clavien-Dindo grade [15]. Within 2 years after discharge, the patients will be followed up every 3 months, and every 6 months after 2 years. The follow-up mainly included blood routine, liver and kidney function, serum CEA, and medical whole abdomen-enhanced CT/MRI. The main clinical outcomes of this study were overall survival (OS) and disease-free survival (DFS).

### Statistical analysis

Patients’ data were retrospectively collected, and statistical analyses were performed using SPSS version 25.0 (SPSS Inc. Chicago, IL, USA). As we identified baseline characteristics mismatching between the two groups after patient grouping, we applied propensity score matching (PSM) analysis to minimize bias caused by non-randomized grouping. The variables selected for the propensity score model are shown in Table 1. The quantitative variables are expressed as mean (SD) if they presented a normal distribution or otherwise as median and range. Qualitative variables are presented in absolute numbers and percentages. Normally distributed continuous data were compared by means of the Student’s *t* test and skewed-distributed by the Mann–Whitney *U* test, and ordinal data were compared in a *χ*^2^ test or Fisher’s exact test. Survival was described using the Kaplan–Meier method, and differences between subgroups were reviewed with the log-rank test. Two-sided *p* values < 0.05 were considered to be statistically significant.

**Table 1:** Baseline characteristics before propensity score matching

Covariates	Time interval (TI) after NAC to surgery		*P* value
	4≦TI < 6 (*n* = 132)	6≦TI≦8 (*n* = 123)	
Sex (M:F)	88:44	76:47	0.417
Age (years)	61.0 (53.3, 65.8)	59 (54, 67)	0.386
Over weight (BMI > 24)	34 (25.8)	28 (22.8)	0.578
Diabetes	21 (15.9)	15 (12.2)	0.395
Hypertension	34 (25.8)	28 (22.8)	0.578
Coronary heart disease	5 (3.8)	7 (5.7)	0.473
Liver cirrhosis	11 (8.3)	7 (5.7)	0.410
NAC cycles	4 (3,5)	4 (3, 5)	0.381
NAC regimes			0.261
FOLFIRI	44 (33.3)	30 (24.4)	
CapeOX	38 (28.8)	37 (30.1)	
FOLFOX	50 (37.9)	56 (45.5)	
Progressive disease during NAC	18 (13.6)	12 (9.8)	0.337
Toxic reactions after NAC	5 (3.8)	15 (12.2)	0.013*
Serum CEA (> 10 ng/mL)	113 (85.6)	105 (85.4)	0.957
Hemoglobin (g/L)	120.2 (96.2, 134.5)	123.0 (102.0, 135.0)	0.259
WBC (10^9^/L)	5.9 (4.5, 7.3)	6.4 (4.7, 7.9)	0.109
PLT (10^9^/L)	111.6 (76.9, 154.2)	113.4 (81.9, 155.7)	0.614
Total bilirubin (μmol/L)	17.9 (12.8, 22.3)	14.6 (11.6, 19.4)	0.004*
Albumin (g/L)	38.8 (34.4, 43.2)	38.1 (33.7, 44.2)	0.659
AST (IU/L)	38.5 (28.3, 54.8)	40.0 (28.0, 60.0)	0.513
ALT (IU/L)	33.0 (22.0, 50.8)	36.0 (23.0, 62.0)	0.317
Primary tumor			
Tumor size (cm)	3.65 (2.7, 4.7)	3.5 (2.5,4.7)	0.449
Tumor location (left:right)	66:66	64:59	0.746
Number of tumors	1 (1, 1)	1 (1, 1)	0.909
Tumor grade (medium/high:low)	66:66	77:46	0.043*
Nerve invasion	39 (29.5)	25 (20.3)	0.090
Cancer nodule	0.5 (0, 1.75)	0 (0, 1)	0.244
Lymph node metastasis	64 (48.5)	53 (43.1)	0.388
Liver metastases			
Tumor size (cm)	3.7 (2.2, 5.0)	3.1 (2.1, 4.2)	0.093
Number of tumors	1 (1, 2)	1 (1, 2)	0.685
Tumor grade (medium/high:low)	75:57	77:46	0.347
Liver capsule invasion	58 (43.9)	37 (30.1)	0.022*
Portal vein tumor thrombus	21 (15.9)	7 (5.7)	0.009*
Vascular tumor thrombus	40 (30.3)	35 (28.5)	0.746
Satellite nodules	22 (16.7)	14 (11.4)	0.226
Extrahepatic invasion	15 (11.4)	9 (7.3)	0.269
Surgical procedure			
ASA grade ≥ 3	3 (2.3)	4 (3.3)	0.632
R0 resection	125 (94.7)	110 (89.4)	0.118
Major liver resection	41 (31.1)	30 (24.4)	0.235

Continuous variables with normal distribution are presented as mean value ± SD while others are presented as median (IQR). Categorical variables are presented as frequency(percentage) unless otherwise stated

*p<0.05

## Result

### Baseline characteristics

**Fig. 1:**
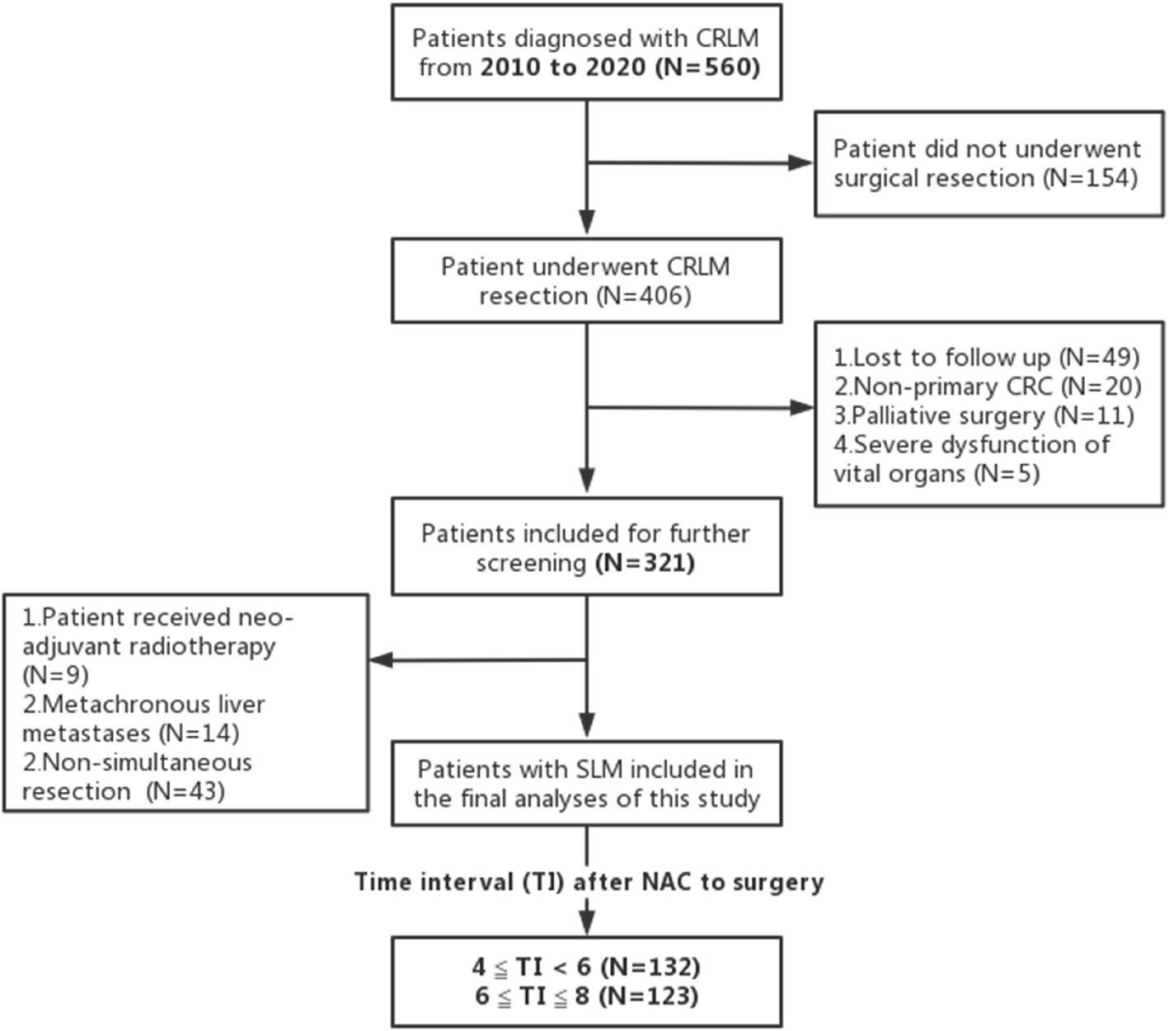
Flow diagram for the selection of patients with SLM included in the final analyses of this study

255 SLM patients who underwent NAC before surgery were finally included. A flow diagram of the included and excluded patients is provided in Fig. 1. We ranked patients according to the time interval (TI) after NAC to surgery from low to high and took the median “6 weeks” as the division basis and divided the patients into two groups (4≦TI < 6, *n* = 132; 6≦TI≦8; *n* = 123). Baseline characteristics in terms of toxic reactions after NAC, total bilirubin, primary tumor grade, liver capsule invasion, and portal vein tumor thrombus showed significant difference before matching. After matching, 94 patients in 4≦TI < 6 group and 94 patients in 6≦TI≦8 group were matched (caliper = 0.2) with all baseline balanced. Tables 1 and 2 shows baseline characteristics between the two groups before and after PSM.

**Table 2:** Baseline characteristics after propensity score matching

Covariates	Time interval (TI) after NAC to surgery		*P* value
	4≦TI < 6 (*n* = 94)	6≦TI≦8 (*n* = 94)	
Sex (M:F)	61:33	60:34	0.879
Age (years)	61 (54, 66)	59 (54, 67.25)	0.435
Over weight (BMI > 24)	22 (23.4)	25 (26.6)	0.613
Diabetes	17 (18.1)	13 (13.8)	0.426
Hypertension	26 (27.7)	20 (21.3)	0.309
Coronary heart disease	4 (4.3)	4 (4.3)	1.000
Liver cirrhosis	7 (7.4)	6 (6.4)	0.774
NAC cycles	4 (3,5)	4 (3,5)	0.597
NAC regimes			0.800
FOLFIRI	24 (25.5)	28 (29.8)	
CapeOX	32 (34.0)	31 (33.0)	
FOLFOX	38 (40.4)	35 (37.2)	
Progressive disease during NAC	11 (11.7)	9 (9.6)	0.636
Toxic reactions after NAC	5 (5.3)	3 (3.2)	0.470
Serum CEA (> 10 ng/mL)	79 (84.0)	78 (83.0)	0.844
Hemoglobin (g/L)	123 (100, 136.8)	121 (99.9, 134.7)	0.581
WBC (10^9^/L)	6.0 (4.8, 7.1)	6.1 (4.6, 8.0)	0.198
PLT (10^9^/L)	118.8 (80.1, 157.5)	117.8 (83.3, 165.3)	0.740
Total bilirubin (μmol/L)	17.3 (11.7, 20.7)	15.4 (12.2, 20.8)	0.782
Albumin (g/L)	38.8 (34.6, 43.4)	38.2 (33.7, 44.3)	0.242
AST (IU/L)	37.5 (28.0, 53.3)	42 (30, 65.3)	0.680
ALT (IU/L)	32.0 (21.8, 50.3)	39 (25, 66)	0.779
Primary tumor			
Tumor size (cm)	3.6 (2.5, 5.0)	3.4 (2.6, 4.5)	0.289
Tumor location (left:right)	53:41	49:45	0.558
Number of tumors	1 (1, 1)	1 (1, 1)	0.651
Tumor grade (medium/high:low)	58:36	59:35	0.880
Nerve invasion	21 (22.3)	19 (20.2)	0.722
Cancer nodule	0 (0, 1)	0 (0, 1)	0.727
Lymph node metastasis	41 (43.6)	39 (41.5)	0.768
Liver metastases			
Tumor size (cm)	3.5 (2.2, 5.0)	3.5 (2.4, 4.5)	0.439
Number of tumors	1 (1, 2)	2 (1, 2.3)	0.095
Tumor grade (medium/high:low)	60:34	61:33	0.879
Liver capsule invasion	35 (37.2)	34 (36.2)	0.880
Portal vein tumor thrombus	7 (7.4)	6 (6.4)	0.774
Vascular tumor thrombus	26 (27.7)	20 (21.3)	0.309
Satellite nodules	12 (12.8)	13 (13.8)	0.830
Extrahepatic invasion	10 (10.6)	8 (8.5)	0.620
Surgical procedure			
ASA grade ≥ 3	3 (3.2)	3 (3.2)	1.000
R0 resection	87 (92.6)	85 (90.4)	0.601
Major liver resection	22 (23.4)	28 (29.8)	0.322

Continuous variables with normal distribution are presented as mean value ± SD while others are presented as median (IQR). Categorical variables are presented as frequency(percentage) unless otherwise stated

### Perioperative and oncologic outcomes

The perioperative outcomes after PSM are shown in Table 3. Patients in 6≦TI≦8 group had significantly longer operative times than those who underwent surgery 4–6 weeks after NAC. (278 min vs. 287.5 min, *p* = 0.015). Patients in 4≦TI < 6 group had significantly higher rates than 6≦TI≦8 group in Clavien-Dindo grade (*p* = 0.003), overall postoperative complications (73.4% vs. 46.8%, *p* < 0.001), overall postoperative infection (34.0% vs. 19.1%, *p* = 0.021), pulmonary infection (24.5% vs. 11.7%, *p* = 0.023), pleural effusion (34.0% vs. 13.8%, *p* = 0.001), and postoperative hemorrhage (13.8% vs. 2.1%, *p* = 0.003).

**Table 3:** Short-term clinical outcomes of patients before and after propensity score matching

Outcomes	Before PSM		*P* value	After PSM		*P* value
	4≦TI < 6 (*n* = 132)	6≦TI≦8 (*n* = 123)		4≦TI < 6 (*n* = 94)	6≦TI≦8 (*n* = 94)	
Operation time (min)	279.5 (251, 295)	287 (268, 303)	0.020*	278 (245.25, 296)	287.5 (272, 305)	0.015*
Intraoperative hemorrhage (ml)	700 (500, 1000)	650 (500, 850)	0.284	725 (500, 1000)	700 (537.5, 850)	0.703
Intraoperative transfusion	50 (37.9)	35 (28.5)	0.111	33 (35.1)	30 (31.9)	0.643
Overall postoperative complications	90 (68.2)	60 (48.8)	0.002*	69 (73.4)	44 (46.8)	< 0.001*
Overall postoperative infection	41 (31.1)	23 (18.7)	0.023*	32 (34.0)	18 (19.1)	0.021*
Sepsis	4 (3.0)	0 (0.0)	0.149	3 (3.2)	0 (0.0)	0.244
Incision infection	5 (3.8)	5 (4.1)	1.000	3 (3.2)	4 (4.3)	1.000
Abdominal infection	6 (4.5)	5 (4.1)	0.850	5 (5.3)	4 (4.3)	1.000
Pulmonary infection	29 (22.0)	14 (11.4)	0.024*	23 (24.5)	11 (11.7)	0.023*
Pleural effusion	39 (29.5)	17 (13.8)	0.002*	32 (34.0)	13 (13.8)	0.001*
Gastrointestinal dysfunction	17 (12.9)	8 (6.5)	0.087	15 (16.0)	7 (7.4)	0.070
Intestinal anastomosis leak	4 (3.0)	3 (2.4)	1.000	3 (3.2)	3 (3.2)	1.000
Liver failure	5 (3.8)	2 (1.6)	0.501	3 (3.2)	2 (2.1)	1.000
Ascites	11 (8.3)	21 (17.1)	0.035*	8 (8.5)	16 (17.0)	0.080
Jaundice	13 (9.8)	3 (2.4)	0.015*	6 (6.4)	3 (3.2)	0.494
Bile leakage	8 (6.1)	14 (11.4)	0.130	5 (5.3)	11 (11.7)	0.117
Postoperative hemorrhage	15 (11.4)	7 (5.7)	0.107	13 (13.8)	2 (2.1)	0.003*
Postoperative hospital stay	7 (6,9)	7 (6,9)	0.868	7 (5.75, 8)	7 (6, 9)	0.307
Clavien-Dindo Grade			0.011*			0.003*
I	28 (21.2)	11 (8.9)		24 (25.5)	9 (9.6)	
II	39 (29.5)	36 (29.3)		28 (29.8)	25 (26.6)	
IIIa	3 (2.3)	3 (2.4)		2 (2.1)	3 (3.2)	
IIIb	12 (9.1)	8 (6.5)		9 (9.6)	5 (5.3)	
IV	7 (5.3)	2 (1.6)		6 (6.4)	2 (2.1)	

Continuous variables with normal distribution are presented as mean value ± SD while others are presented as median (IQR). Categorical variables are presented as frequency (percentage) unless otherwise stated

*p<0.05

**Fig. 2:**
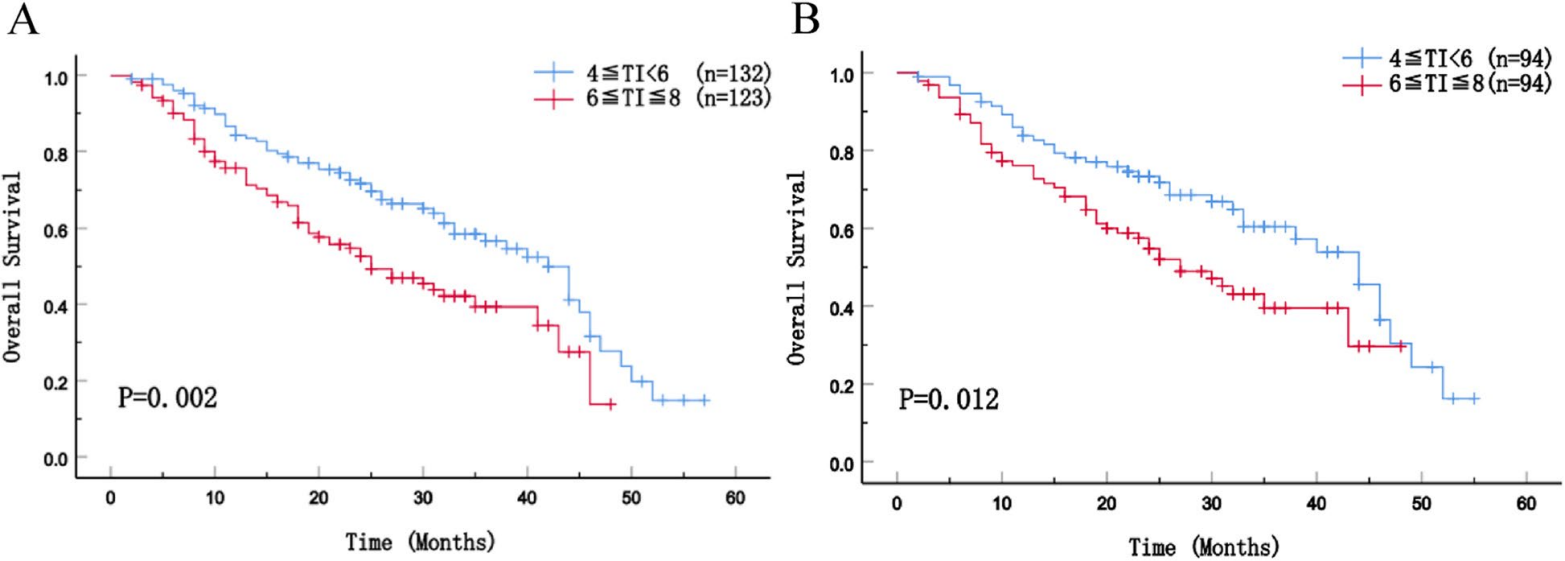
Overall survival of patients before PSM (**A**) and after PSM (**B**)

**Fig. 3:**
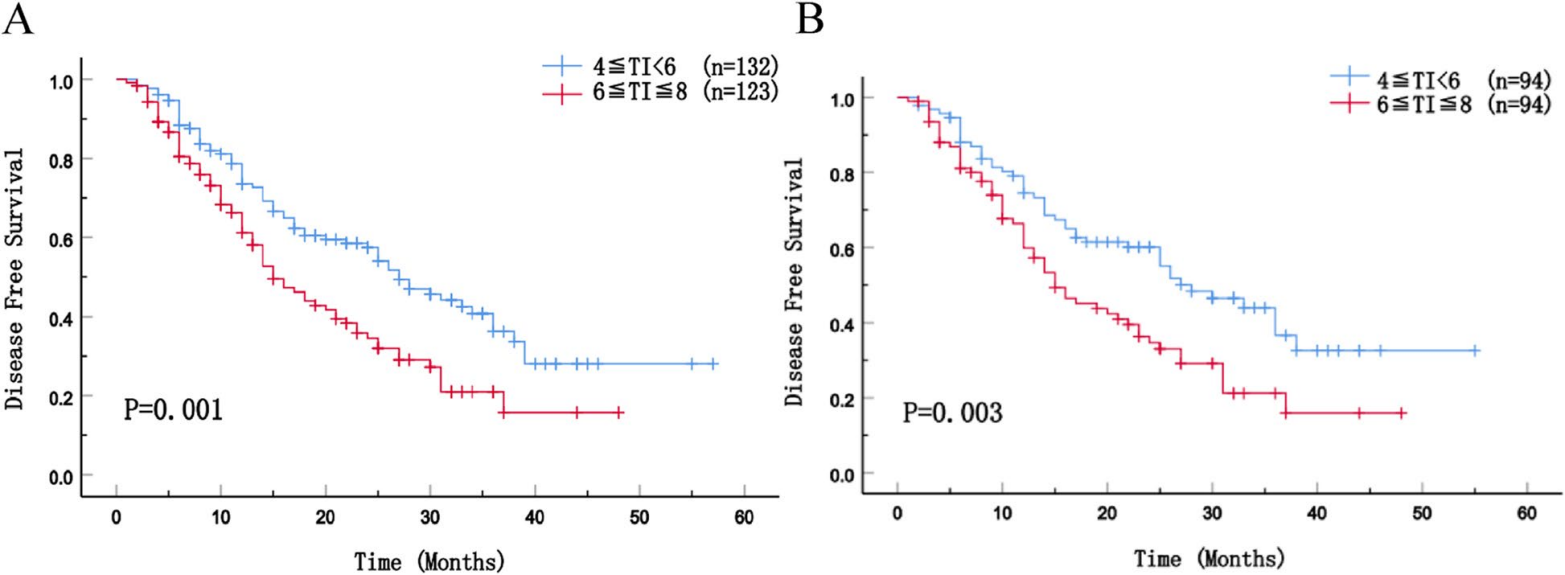
Disease-free survival of patients before PSM (**A**) and after PSM (**B**)

During the follow-up period (median duration was 28 months), 145 (56.9%) patients experienced tumor recurrences, and 126 (49.4%) patients were dead. The median OS of patients in 6≦TI≦8 group was 25 months before PSM and 27 months after PSM, which was significantly worse than that of patients who underwent surgery 4–6 weeks after NAC (42 months and 44 months) (Fig. 2A, *p* = 0.002; Fig. 2B, *p* = 0.012). The cumulative overall survival rates of patients in 6≦TI≦8 group at 1, 2, and 3 years after curative-intent resection were 76.1, 57.5, and 39.5%, respectively, which were significantly lower than those of patients in 4≦TI < 6 group. The median DFS was better for patients who underwent surgery 4–6 weeks after NAC (before matching: 27 months vs. 15 months; after matching: 28 months vs. 15 months) (Fig. 3A, *p* = 0.001; Fig. 3B, *p* = 0.003).

Subgroup analysis found that the above conclusions still apply in age ≥ 60, non-anemic patients and patients who underwent R0 resection. Both OS and DFS in these patients were negatively correlated with TI. OS was inversely correlated with TI in patients without preoperative jaundice. DFS was negatively correlated with TI in patients with preoperative jaundice (Table 4).

**Table 4:** Subgroup analysis of OS and DFS in 255 patients with SLM after NAC

Clinical indicators	Time interval (TI) after NAC to surgery (no. of patients)	OS (mean survival time)	*P* value	DFS (mean disease-free time)	*P* value
Age					
Age < 60	4≦TI < 6 (*N* = 61)	35.94 months	0.055	29.43 months	0.113
	6≦TI≦8 (*N* = 64)	28.35 months		21.73 months	
Age ≥ 60	4≦TI < 6 (*N* = 71)	35.37 months	0.011*	27.93 months	0.002*
	6≦TI≦8 (*N* = 59)	26.22 months		18.91 months	
Chronic disease					
Yes	4≦TI < 6 (*N* = 52)	37.46 months	0.012*	28.64 months	0.019*
	6≦TI≦8 (*N* = 46)	28.45 months		20.25 months	
No	4≦TI < 6 (*N* = 80)	34.50 months	0.045*	29.29 months	0.014*
	6≦TI≦8 (*N* = 77)	27.74 months		20.34 months	
Anemia (M:Hb < 120 g/L,F:Hb < 110 g/L)					
Yes	4≦TI < 6 (*N* = 64)	35.75 months	0.052	25.98 months	0.063
	6≦TI≦8 (*N* = 45)	26.39 months		19.29 months	
No	4≦TI < 6 (*N* = 68)	36.16 months	0.030*	31.63 months	0.007*
	6≦TI≦8 (*N* = 78)	27.49 months		20.32 months	
Preoperative jaundice					
Yes	4≦TI < 6 (*N* = 73)	36.39 months	0.062	30.94 months	0.001*
	6≦TI≦8 (*N* = 40)	27.50 months		16.92 months	
No	4≦TI < 6 (*N* = 59)	34.18 months	0.036*	26.63 months	0.069
	6≦TI≦8 (*N* = 83)	27.50 months		22.92 months	
Exclude non-R0 resection	4≦TI < 6 (*N* = 125)	35.44 months	0.007*	29.45 months	0.002*
	6≦TI≦8 (*N* = 110)	27.88 months		20.27 months	

*p<0.05

### Univariate and multivariate analyzes

Univariate and multivariate analyzes showed that TI after NAC to surgery was significantly correlated with both OS and DFS of patients, and the longer the TI, the worse the prognosis. The number of primary tumors is risk factors for OS. Vascular tumor thrombus of liver metastases and gastrointestinal dysfunction were risk factors for DFS (Table S1).

## Discussion

There is still controversy in clinical practice regarding the appropriate surgery interval for SLM patients after NAC, and different guidelines recommend different timing for surgery. For example, the latest version of the National Comprehensive Cancer Network (NCCN) updated guidelines for the management of metastatic colorectal cancer recommends that the surgical interval after NAC be 5–12 weeks [16], while the “ESMO Guidelines” recommends that the optimal operation time is 6–8 weeks after NAC [17]. Apart from the ongoing controversy, these guidelines have a broad coverage of CRLM patient population, but fail to address the differences within the CRLM patient population, which inevitably leads to heterogeneity among the groups included in the studies. For example, synchronous liver metastasis (SLM) and metachronous liver metastasis (MLM) patients have different prognostic characteristics [4, 5]. SLM patients have a worse prognosis. Therefore, the applicability of these guidelines for patients with SLM remains to be proven. Likewise, several studies have been devoted to the optimal timing of surgery for patients with CRLM after NAC. For example, Chen et al. found that patients with a longer time to surgery (TTS) were more likely to have adverse pathological responses, while those with a shorter TTS had significantly better PFS and OS [18]. Thomas et al. found that compared with CRLM patients with time to resection (TTR) < 2 months, patients with TTR ≥ 2 months had shorter RFS and OS [19]. Although these studies indicate that different surgical timings after NAC can affect patient outcomes, they also do not pay attention to the differences in prognosis among different subtypes of CRLM patients. In addition, these studies do not address the impact of different timing of surgery on postoperative complications in CRLM patients. Therefore, we conducted this study on initially resectable SLM patients to investigate the optimal timing of surgery and provide the greatest survival time for these patients.

In present study, SLM patients were divided into early resection subgroup (4 ≤ TTS < 6) and delayed resection subgroup (6 ≤ TTS ≤ 8) according to the TTS after NAC. Based on data from the current study, patients in the early surgical resection subgroup had better OS and DFS than the delayed surgery subgroup. In response to this finding, and in conjunction with previous studies in other malignancies, we can clarify that some of the following reasons may contribute to the worse prognosis of patients who delay underwent surgery. First of all, not all SLM patients receiving NAC can benefit from delayed surgical resection, and some patients with insignificant or enlarged lesions after NAC may cause further tumor growth and metastasis due to delayed resection [20]. Second, severe complications during NAC are a key factor in increasing TTS, and the low physical status of these patients may be directly related to poor prognosis [21]. More importantly, more studies have shown that more than 6 weeks after NAC leads to the regrowth of potentially resistant tumor cell populations while further reducing the efficacy of surgical treatment [22, 23]. The present study matched the two groups of patients at baseline level after propensity score (including the progressive disease during NAC and the incidence of serious complications after NAC); therefore, we believe that this difference in prognosis may be more related to the biological characteristics of the tumor itself. Undergoing radical surgical resection within 4–6 weeks after NAC may be a better option.

Notably, among SLM patients who underwent radical resection earlier, there may still be some patients who have not recovered from the post-NAC neutropenic window, and premature surgery may theoretically lead to an increased rate of postoperative complications, especially those related to postoperative infection [24]. In addition, the liver damage caused by NAC cannot be fully recovered in a short time, and therapeutic liver resection at this time will further increase the burden on the liver [25]. Therefore, this study found that the incidence of postoperative jaundice in the 4≦TI < 6 group was higher than that in the 6≦TI≦8 group. Meanwhile, the increase in the incidence of postoperative hemorrhage in the 4≦TI < 6 group may also be related to the transient disorder of coagulation function caused by liver function decline.

Due to differences in postoperative complications between the two groups of patients, further investigation was conducted to explore the impact of postoperative complications on the prognosis of SLM patients undergoing simultaneous resection after NAC. Initially, we classified postoperative complications in both groups using the Clavien-Dindo grade system and found that the main differences were concentrated in grade I complications. We further analyzed the factors related to postoperative complications using single-factor and multi-factor analyses, but found no correlation between postoperative complications and prognosis. This result further confirms the viewpoint that tumor biology, rather than postoperative course, strongly determines the probability of patient survival, as reported in previous studies [26, 27]. In summary, patients with SLM may be a better choice for surgery within 4–6 weeks after receiving NAC. Although patients with SLM undergoing surgery 4–6 weeks after NAC has a higher rate of postoperative complications, radical surgery is still recommended for a better survival benefit.

Our study is the first to investigate the timing of surgery after NAC in resectable SLM patients. In addition to focusing on the prognosis (OS, DFS) of patients, we also paid attention to the short-term clinical outcomes of patients after surgery. Meanwhile, this study performed propensity score matching on the two groups of patients, minimizing the impact of retrospective study bias on the conclusions. This study can provide new evidence for clinical diagnosis and treatment. However, this study still has the following limitations. The first point to consider is that this study is a retrospective cohort study, which unavoidably introduces bias. Next, the relatively special population included in this study led to a small sample size. Thirdly, this study is a single-center cohort study, and the conclusions may not necessarily represent the situation in other countries and regions. Therefore, future randomized controlled studies and large-scale multicenter prospective cohort studies are needed for further verification.

## Conclusions

Based on data from the current study, patients in the early surgical resection subgroup had better OS and DFS than the delayed surgery subgroup. Patients with SLM may be a better choice for surgery within 4–6 weeks after receiving NAC. Although patients with SLM undergoing surgery 4–6 weeks after NAC has a higher rate of postoperative complications, radical surgery is still recommended for a better survival benefit.

### Supplementary Information

**Additional file 1: Table S1.** Univariate and multivariate analysis of prognostic factors in 255 patients with SLM after NAC.

## Data Availability

All data are from West China Hospital of Sichuan University, and the original data involved in the article can be obtained from the corresponding author.

## Abbreviations

ALB: Albumin
ALT: Alanine aminotransferase
ASA: American Society of Anesthesiologists
AST: Aspartate aminotransferase
CapeOX: Capecitabine plus oxaliplatin
CEA: Carcinoembryonic antigen
CRC: Colorectal cancer
CRLM: Colorectal cancer liver metastases
DFS: Disease-free survival
FOLFIRI: Fluorouracil, leucovorin, and irinotecan
FOLFOX: Fluorouracil, leucovorin, oxaliplatin
FOLFOXIRI: Fluorouracil, leucovorin, oxaliplatin, and irinotecan
Hb: Hemoglobin
LARC: Locally advanced rectal cancer
MLM: Metachronous liver metastases
NAC: Neoadjuvant chemotherapy
NCCN: National Comprehensive Cancer Network
OS: Overall survival
PCR: Pathologic complete response
PLT: Platelets
PSM: Propensity score matching
RFS: Recurrence-free survival
SLM: Synchronous liver metastases
TB: Total bilirubin
TI: Time interval
TTS: Time to surgery
WBC: Leukocyte
