# Supplementary material for: Timing of surgery in patients with synchronous colorectal cancer liver metastases undergoing neoadjuvant chemotherapy: a propensity score analysis
Source: World J Surg Oncol. 2023 Sep 1;21:276. doi: 10.1186/s12957-023-03162-y (PMC10472641; doi:10.1186/s12957-023-03162-y)
Supplement: Supplementary file 1 — Additional file 1: Table S1. Univariate and multivariate analysis of prognostic factors in 255 patients with SLM after NAC. [file 12957_2023_3162_MOESM1_ESM.docx]

TableS1. Univariate and multivariate analysis of prognostic factors in 255 patients with SLM after NAC.

| Variable | HR (95% CI) | Univariate analysis p-value | HR (95% CI) | Multivariate analysis p-value |
| --- | --- | --- | --- | --- |
| **OS** |  |  |  |  |
| Time interval (TI) after NAC to surgery | 1.74(1.22, 2.50) | 0.003* | 1.65(1.14, 2.38) | 0.007* |
| Diabetes | 0.61(0.36, 1.05) | 0.077 |  |  |
| Primary tumor |  |  |  |  |
| --Number of tumors | 3.39(0.72, 15.86) | 0.121 | 2.96(1.11, 7.89) | 0.030* |
| --Cancer nodule | 0.85(0.68, 1.05) | 0.130 |  |  |
| Liver metastases |  |  |  |  |
| --Tumor size | 0.78(0.60, 1.01) | 0.056 |  |  |
| --Tumor grade | 1.41(0.98, 2.03) | 0.064 |  |  |
| Major liver resection | 0.71(0.47, 1.08) | 0.106 |  |  |
| Intraoperative hemorrhage | 0.68(0.52, 0.90) | 0.007* | 0.99(0.99, 1.00) | 0.033* |
| **DFS** |  |  |  |  |
| Time interval (TI) after NAC to surgery | 1.72(1.24, 2.39) | 0.001* | 1.65(1.18, 2.31) | 0.003* |
| ALT | 1.13(1.00, 1.29) | 0.058 |  |  |
| Primary tumor |  |  |  |  |
| --Tumor location | 0.7(0.50, 0.97) | 0.033* |  |  |
| Liver metastases |  |  |  |  |
| --Tumor size | 0.82(0.66, 1.04) | 0.099 |  |  |
| --Vascular tumor thrombus | 1.44(1.02, 2.04) | 0.038* | 1.54(1.08, 2.19) | 0.016* |
| Major liver resection | 0.6(0.41, 0.88) | 0.009* |  |  |
| Intraoperative hemorrhage | 0.73(0.57, 0.93) | 0.011* |  |  |
| Gastrointestinal dysfunction | 1.57(0.93, 2.65) | 0.091 | 1.71(1.01, 2.89) | 0.046* |
| Liver failure | 0.3(0.07, 1.20) | 0.088 |  |  |
